# Positive Association of Physical Activity with Both Objective and Perceived Measures of the Neighborhood Environment among Older Adults: The Aichi Workers’ Cohort Study

**DOI:** 10.3390/ijerph17217971

**Published:** 2020-10-29

**Authors:** Yuanying Li, Hiroshi Yatsuya, Tomoya Hanibuchi, Atsuhiko Ota, Hisao Naito, Rei Otsuka, Chiyoe Murata, Yoshihisa Hirakawa, Chifa Chiang, Mayu Uemura, Koji Tamakoshi, Atsuko Aoyama

**Affiliations:** 1 Department of Public Health, Fujita Health University School of Medicine, Aichi 470-1192, Japan; yatsuya@fujita-hu.ac.jp (H.Y.); ohtaa@fujita-hu.ac.jp (A.O.); naitoh@fujita-hu.ac.jp (H.N.); 2 Department of Public Health and Health Systems, Nagoya University Graduate School of Medicine, Aichi 466-8550, Japan; y.hirakawa@med.nagoya-u.ac.jp (Y.H.); keihatsu@med.nagoya-u.ac.jp (C.C.); mayu-u@med.nagoya-u.ac.jp (M.U.); aaoyama@nuas.ac.jp (A.A.); 3 Graduate School of Environmental Studies, Tohoku University, Sendai 980-8572, Japan; hanibuchi@gmail.com; 4 Section of NILS-LSA, National Center for Geriatrics and Gerontology, Aichi 474-8511, Japan; otsuka@ncgg.go.jp; 5 Department of Social Science, National Center for Geriatrics and Gerontology, Aichi 474-8511, Japan; cmurata@ncgg.go.jp; 6 Department of Nutrition, Tokai Gakuen University, Aichi 468-8514, Japan; 7 Department of Nursing, Nagoya University School of Health Science, Aichi 461-8673, Japan; tamako@met.nagoya-u.ac.jp; 8 Nagoya University of Arts and Sciences, Aichi 481-8503, Japan

**Keywords:** built environment, geographic information systems, perception, physical activity, older adults, observational study

## Abstract

We examined the association between objective and perceived neighborhood characteristics and self-reported leisure-time physical activity (PA) in older Japanese residents living in areas ranging from metropolitan to rural in 2016. Objective measures used were walkability and the numbers of parks/green spaces and sports facilities within 500 or 1000 m of subjects’ homes, calculated using geographic information systems. Subjective measures were the subjects’ perceptions of their neighborhoods, assessed using a structured questionnaire. All variables were divided into three groups, and the lowest tertile was used as the reference. We assessed the location and frequency of strolling or brisk walking, moderate-intensity PA, and vigorous-intensity PA (sports) using a self-reported questionnaire and defined as performing a certain type of PA 3–4 times/week as a habit. Living in a neighborhood in the highest tertile for walkability and number of parks/green spaces as well as perception of having good access to recreational facilities, observing others exercising and the presence of walkable sidewalks was associated with walking and sports habits (multivariable odds ratios (ORs): 1.33–2.46, all *p* < 0.05). Interestingly, objective measures of PA-friendly environmental features were inversely associated with moderate-intensity PA habits, potentially because moderate-intensity PA consisted predominantly of gardening. In conclusion, living in an environment supportive of PA, whether objectively or subjectively measured, is related to leisure-time PA habits among older Japanese adults.

## 1. Introduction

The benefits of regular physical activity (PA) are well established, however; a large proportion of the world’s population does not meet the current recommendations for adequate PA, especially among older adults [1,2,3]. Improving PA through exercise is of greater relevance to older adults, as moderate- to vigorous-intensity physical activity (MVPA) substantially decreases after retirement [4].

Improvements to residential environments with the goal of promoting PA has received growing attention. The residential neighborhood may be particularly important for older adults, as they spend more time around the home. Older adults are also more vulnerable to mobility barriers due to a generally decreased physical function compared to their younger counterparts. Thus, public health researchers have become interested in the influence of the built environments surrounding older individuals on their PA. A recent meta-analysis [5] comprehensively evaluated a variety of environmental features in relation to PA in older adults; however, the majority of the studies were conducted in Western countries. Considering that city designs, and social norms may differ substantially between countries, findings derived from Western countries may not be fully generalizable to other locations. To date, associative studies based on East Asian countries, especially those using objectively measured environmental information [6,7,8], are scarce.

Additionally, many previous studies examined the relationship with overall PA, which may include PA irrelevant to the targeted environmental characteristics. Moreover, the latest PA guidelines for adults proposed recommendation separately for different levels of intensity [9,10]; however, specific associations were insufficiently studied [5]. Thus, the present study included 1601 subjects of local government retirees who resided in areas with great variability of geographical setting and environmental attributes, and it considered exercise-domain walking, moderate-intensity PA, and vigorous-intensity PA separately to address possible differences between the different types of PA in terms of their association with environmental characteristics. To better understand the overall observations, a random sample of approximately 300 subjects from the initial study population was taken and followed up for two years to extract details of PA and the venues where it was performed.

The aim of the present study was thus to explore the effects of geographic information system (GIS)-measured walkability and density of parks/open spaces and sports facilities, as well as subjective measures of the built and social environments around individuals’ residences, on different types of PA among government retirees, who are expected to be relatively homogenous in terms of socioeconomic status (SES).

## 2. Materials and Methods

### 2.1. Subjects and Study Location

The Aichi Workers’ Cohort Study, initiated in 1997, is an ongoing epidemiologic study on non-communicable diseases, including diabetes and cardiovascular disease. The subjects are current employees and retirees of the local government of Aichi prefecture, located in central Japan. Based on the 2015 Japanese Census, the Aichi prefecture consists of 7.48 million people with a density of 1449 people/km^2^, and the percentage of individuals 65 years or older is 23.8%, lower than the national average of 26.7% [11]. The capital of the prefecture, Nagoya city, is the fourth-largest metropolitan city in terms of population in Japan [12].

The present study used data from individuals who retired on or prior to 31 May 2016. These retirees gave written consent and indicated a willingness to respond to post-retirement surveys by providing their residential address for postal mail. A self-administered questionnaire was distributed in November 2016 and collected via post. The questionnaire included respondents’ perception of their built neighborhood environments, leisure-time PA, depression status, medical history, and other lifestyle features. Among 2564 (2102 men and 462 women) subjects initially surveyed, 2066 (1711 men and 355 women) responded. We excluded those younger than 60 years of age and those missing data, leaving 1601 subjects (1358 men and 243 women) for the analysis.

The study protocol was approved by the Ethics Review Committee of Nagoya University School of Medicine (2007-0504) and Fujita Health University (HM17-470 and HM19-018).

### 2.2. Objective Environmental Measures

We objectively measured three environmental indices: the availability of parks/green spaces, availability of sports facilities, and walkability. The detail of the walkability index in Japan has been described elsewhere [13,14]. Briefly, the walkability index consists of population density, road density, access to parks, and access to retail areas, reflecting friendliness of the built environment towards walking. As an objective unit of neighborhood boundaries, we employed chocho-aza, the smallest administrative unit. All of our data were based on National Land Numerical Information (NLNI) by Ministry of Land, Infrastructure, Transport and Tourism of Japan, the 2010 Population Census of Japan [15], and retail area data by Zenrin Co. Ltd. (Kitakyushu-shi, Fukuoka, Japan) as of 2011. ArcGIS 10.3 (ESRI, Redlands, CA, USA) was used to assess the number of parks/green spaces and sports facilities within a 500 and 1000 m radius of the individual’s residence based on the street network (i.e., the network radial buffer), to facilitate comparison with previous studies where these two scales were most commonly used [5,6,8]. Data on parks/green spaces were also obtained from NLNI. The locations of public or commercial sports/recreational facilities, such as sports centers, gyms, or fitness facilities (hereafter collectively referred to as sports facilities) were based on point data from the Yellow Pages telephone directory of businesses.

### 2.3. Perceived Environmental Measures

International Physical Activity Questionnaire Environmental Module (IPAQ-E) consisting of 17 questions was applied to measure the perceived neighborhood environment related to PA [16,17]. The Japanese version of IPAQ-E comprising seven core items and four recommended items with concurrent validity and test-retest reliability has been demonstrated as being effective among Japanese adults [18]. In the present study, we arbitrarily selected five environmental features: (1) good access to public transportation, (2) good access to recreational facilities, (3) presence of walkable sidewalks, (4) observing others exercising (social environment), and (5) poor traffic safety. The following four response options were available: strongly disagree, somewhat disagree, somewhat agree, and strongly agree. The test-retest reliability with respect to five individual question items in terms of Spearman’s correlation ranged from 0.82 to 0.85 and Kappa statistics ranged from 0.67 to 0.79 [18].

### 2.4. Definition of Regular Recreational Physical Activity

The participants were asked to estimate their average frequency and duration of four types of recreational PA in the past year: (1) strolling, (2) brisk walking, (3) moderate-intensity PA such as golf, “gate ball”, a Japanese game similar to croquet, or gardening, and (4) vigorous-intensity PA such as tennis, jogging, aerobics, or swimming. Two options for PA location were also given: (1) home or neighborhood or (2) away from neighborhood.

The frequency of PA was self-reported separately on a five-point scale: almost none, 1–3 times/month, 1–2 times/week, 3–4 times/week, and almost every day. Bout-duration of each physical activity was assessed on a six-point scale: less than 30 min, 30–59 min, 1 to <2 h, 2 to <3 h, 3 to <4 h, or more than 4 h.

Individuals who responded that they had engaged in strolling or brisk walking, moderate-intensity PA, or vigorous-intensity PA (hereafter referred to as sports) for three–four times or more per week were defined as having a walking habit, moderate-intensity PA habit, or sports habit, respectively.

### 2.5. Types of Moderate-Intensity PA and Location

In order to better understand the negative associations of objective environmental measures with moderate-intensity PA, we carried out an additional interview survey with some of the participants. Initially, a randomly selected 730 subjects were invited to a follow-up health check-up and fitness survey in 2018. Two hundred and eighty-five older adults attended the survey, and returned the self-reported questionnaire on PA, which was sent to subjects’ home address one month prior to the survey. They were asked to fill in the questionnaire and bring to the survey site. The interview was conducted by trained staff. They asked the participants an open question about the content and the venue of the PA they performed if they had self-reported in the questionnaire that they do moderate-intensity PA 3–4 times per week or more.

### 2.6. Statistical Analysis 

Objective environmental variables were categorized into tertiles in order to describe potential non-linear relationship. The following cutoff values were used: for walkability index: 4–25 (reference), 26–32, or 33–40; the number of parks/green spaces within a 500 m radius of the respondent’s home: 0 (reference), 1, or 2–9; and within a 1000 m radius: 0–1 (reference), 2–6, or 7–21; the number of sports facilities within a 500 m radius: 0 (reference), 1, or 2–10; and within a 1000 m radius: 0 (reference), 1–2, or 3–31. We also examined these associations using continuous environmental variables.

Perceived environment variables, except for those regarding poor traffic safety, were also divided into the following three groups: negative group (strongly disagree and somewhat disagree, collapsed because the number of cases in either category was extremely small (e.g., 0 or less than 10) for some items), positive (somewhat agree), and very positive (strongly agree). The response rating for poor traffic safety used reverse scoring.

Strolling or brisk walking, moderate-intensity PA, and sports were graded as either ‘engaging in 3‒4 times or more per week’ or ‘not’.

The multivariable model used was adjusted for age, sex, the number of people in the household (alone, two people, three or more people), body mass index based on self-reported body weight and height (<21, 21 to <25, or ≥25 kg/m^2^), smoking status (never, former, or current), alcohol drinking habits (never, former, or current), sleeping hours (<7, 7 to <8, or ≥8 h), history of cardiovascular disease (yes, no), history of hypertension or metabolic disorders (yes, no), depression status assessed by the 11-item Center for Epidemiologic Studies Depression Scale exceeding 7 points (yes, no), and the number of motor vehicles in the household (0, 1, ≥2).

A single-level binomial logistic regression model was applied. Age-adjusted and multivariable-adjusted models were constructed to obtain odds ratios (ORs) and their 95% confidence intervals. Given the multilevel structure of the data, which comprised individuals (level 1) nested within municipalities (city/ward/town/village) of residence (level 2), we initially fitted the data to a two-level multilevel model. There was no significant municipality-level variation in walking habit (Z = 0.88, *p* = 0.19) or sports habit (Z = 0.27, *p* = 0.39) when setting the municipalities of residence as a random effect in the unconditional model. However, there was a statistically significant amount (5.5%) of variation in moderate-intensity PA habit that was explained by municipality (Z = 2.02, *p* = 0.02). The results derived from the multilevel model were similar to those derived from a single-level binomial regression model. We therefore employed a single-level model for the present study.

Additional analyses were done by redefining respective PA habits to include only subjects whose PA durations were at least 30 min per bout.

Analysis using variables obtained in the interview was done by simply calculating percentages of the activity/venue in individuals with moderate-intensity PA three‒four times per week or more. 

*p* values < 0.05 and < 0.10 (two-tailed) were considered to be statistically significant and marginally significant, respectively. All analyses were performed using the software SAS (Statistical Analysis Software 9.4, SAS Institute Inc, Cary, North Carolina, USA).

## 3. Results

A total of 85% of the subjects were male. The mean (standard deviation) age was similar for men and women: 67.9 (3.8) and 67.6 (4.0), respectively. A total of 44.7%, 18.4%, and 6.2% of subjects had walking habits, moderate-intensity PA habits, and sports habits, respectively. A majority of subjects who reported walking habits reported that they did so within the neighborhood: 80% for habitual stroll and 61% habitual brisk walking. The corresponding proportions of habitual moderate-intensity PA and sports were 73% and 35%, respectively (Table 1).

Walking habits related positively with a walkability index of 33–40, 2–9 parks/green spaces (500 m), and 3–31 sports facilities (1000 m) with multivariable ORs ranging from 1.33–1.44 (*p* < 0.05). There was a positive relationship (multivariable ORs ranging from 1.33–1.83 (*p* < 0.05)) between walking habits and perceptions of good access to recreational facilities, observing others exercising, and presence of walkable sidewalks (Table 2).

For moderate-intensity PA, the multivariable ORs (highest tertile versus lowest) ranged from 0.51 to 0.69 (*p* < 0.05) for walkability, the numbers of parks or green spaces (500 m and 1000 m), and numbers of sports facilities (1000 m) (Table 3). There were no significant associations between perceived features and habitual moderate-intensity PA (Table 3).

For sports habits, the multivariable ORs for association with walkability indices of 26‒32 and 33‒40, 1 and 2‒9 parks/green spaces (500 m), and 7‒21 parks/green space (1000 m) ranged from 1.74–2.06 (*p* < 0.05). Subjects with positive or very positive perceptions of good access to recreation facilities and presence of walkable sidewalks, as well as with very positive perception of observing others exercising as features of the neighborhood environment, showed multivariable ORs for sports habits ranging from 1.76–2.46 (*p* < 0.05) (Table 4).

An additional analysis taking exercise duration (i.e., 30 min or more per bout) into consideration revealed similar results as the main findings (data not shown). Of the 285 subjects who completed interview surveys in 2018, 155 reported engaging in moderate-intensity PA three‒four times or more per week. Of the 155 subjects, 84% reported courtyard gardening. Other moderate-intensity PA included golf in the neighborhood (7%) and golf outside of the neighborhood (9%).

## 4. Discussion

We found that objective and perceived PA-friendly environmental features were positively associated with walking and sports habits in older Japanese individuals. These associations were independent of a number of confounding variables including medical history, depression, and the number of cohabitants and cars owned by households.

In contrast, objective environmental features were negatively associated with moderate-intensity PA. Our follow-up interview survey, which revealed that moderate-intensity PA consisted mainly of courtyard gardening, may explain these findings. Small numbers of parks/green spaces and recreational facilities as well as low walkability would be characteristic of places with enough space to allow for gardening at home. This is consistent with a previous study that defined gardening more as a home-based work than leisure exercise [19]. Moreover, unlike other exercises that people usually perform voluntarily, courtyard gardening may, like their routine housework, be a necessary activity. The association found in the present study could not be generalized to other moderate-intensity PA outside the home; however, it is encouraging to find that gardening may be an alternative option for subjects living in areas with less PA amenities.

The positive association of walkability with walking habits in the present study is supported by findings on the daily total walking of older adults in Japan [8] as well as leisure walking in the US [20,21,22,23], but inconsistent with a Belgian study on leisure walking [24]. Previous Belgian and American studies reported that walkability was positively associated with total MVPA [20,24] but not with self-reported recreational MVPA [24] among older adults. In the present study, the positive association between walkability and sports habits did not vary notably according to the location of the PA, i.e., whether the PA was performed in the home neighborhood (*n* = 35) or outside it (*n* = 64; data not shown), suggesting that a higher walkability index may promote sports participation by providing easy access to sports venues by walking or other more active forms of locomotion.

Our results showed strong links between the objective density of parks/open spaces and walking and sports habits in older adults. This was supported by findings from a Hong Kong study in which the presence of parks within a neighborhood was associated with a greater amount of leisure walking and other PA [7]. In addition, a higher density of parks was associated with frequency of sports in older adults in Japan [6] and daily total MVPA in the US [25]. Other studies using alternative measures such as area or park accessibility also identified positive effects on leisure walking among older adults in the US [26] and leisure-time PA including walking in Portugal [27] and England [28]. The density of parks was not associated with leisure walking in a US study [25], which may be due to different participant characteristics or different methodologies used by the studies.

In the present study, the expected positive associations between the number of sports facilities and walking and sports habits was only observed when facilities were further away (1000 m). This is comparable to several previous US studies of older adults, in which the density of recreational facilities within a greater distance (1.0 mile or 1–5 miles) of home, but not within smaller distances (500 m or 0.5 mile), exhibited significant positive effects on leisure walking [25], sports [29], total MVPA [25], or leisure PA including walking [30]. In addition to accessibility, use of sports facilities tended to be related to more complex factors, such as the types of sports or activities offered, fees for their use, and safety [31,32]. Therefore, the diversity of sports facilities available may be as important as their proximity [5,31]. We found only a weak association between the density of sports facilities within 1000 m and sports habits, which may be partly explained by the relatively high SES background of the study subjects, who have high rates of car ownership and who may view cost as a low barrier for accessing the more remote sports facilities they prefer [33]. Future studies may collect qualitative aspects of sports facilities and further test for heterogeneity of the associations with specific sports according to individual level of SES.

In general, the associations of density of parks or sports facilities within a 500 m buffer with walking or sports habits were less concordant compared to the associations using a 1000 m buffer, which were at a positive linear direction uniformly. This might suggest that more environmental resources in the immediate neighborhood do not add much to promote PA at the population level. An alternative explanation could be related to the fact that high density of PA amenities in the area is related to high population density, implying the possibility of competition for using those amenities [32]. In any case, efforts to allocate optimal resources should pay attention to the present findings given limited resources that could be used for improving or maintaining the built environment.

IPAQ-E has frequently been applied in East Asian studies and demonstrates generally consistent results with respect to leisure PA outcomes. Our results corroborated earlier findings from Japan [34,35], Hong Kong [36], and Taiwan [37] in which older adults who perceived good access to recreation facilities [36,37], presence of sidewalks [36,37], and observing others exercising [34,37] were more likely to walk for recreation [7,34,37] as well as participate in recreational MVPA other than walking [35]. However, one Japanese study showed no evidence of an association between perceived good access to recreation facilities or presence of sidewalks with leisure walking [34].

Neither recreational walking nor recreational MVPA other than walking were related to perceived good access to public transportation [34,35,37] or traffic safety [25,28,34,35,36,37,38] in early studies of East Asian or Western settings. Similarly, we did not find any significant associations for these two perceived characteristics with walking, moderate-intensity PA, or sports habits.

Our study was unique in that the subjects were retired civil servants, which served to reduce the confounding effects associated with SES [36,39,40]. Objective features that were quantified by different geographic scales and environmental features were linked with different types of PA. Despite the small size of the sub-sample, we extracted the details and locations of PA, which reinforced interpretation of our findings. In addition, we were able to statistically adjust for important potential confounding factors that were often unavailable in studies of geographical characteristics and PA, such as depression symptoms [20], and the number of motor vehicles in the household [41].

There are some limitations that warrant consideration. First, the cross-sectional nature of the present study may suffer from reverse causality; people who prefer exercise may select PA-friendly neighborhoods [32,42]. Second, PA was self-reported only once without considering seasonal variation [43]. However, the non-differential misclassification of weekly frequency would reduce the magnitude of this association [44]. In addition, the use of a questionnaire allowed us to focus on the exercise-driven PA that was relevant to a neighborhood environment. Finally, the subjects of the present study were exclusively civil servant retirees; thus, the generalizability of the present findings to broader community populations may be limited.

## 5. Conclusions

Living in a PA-supportive environment, where support was either objectively measured or subjectively perceived, was related to the leisure-time walking and sports habits of older Japanese adults.

## Figures and Tables

**Table 1:** Characteristics of retired civil servants, Aichi Workers Cohort, 2016.

Characteristics	Men	Women	All
Number of subjects	1358	243	1601
Age, mean (standard deviation, range)	67.9 (3.8, 60–81)	67.6 (4.0, 60–79)	67.9 (3.9, 60–81)
Habitual exercise: more than 3–4 times/week (%)			
Strolling Brisk walking Either strolling or brisk walking Both strolling and brisk walking Moderate-intensity physical activity Vigorous-intensity physical activity	30.5	26.8	29.9
	22.8	18.5	22.2
	45.8	38.3	44.7
	7.5	7.0	7.4
	18.2	19.8	18.4
	5.7	9.1	6.2
Percent of habitual exercise conducted at home or within the home neighborhood (%)			
Strolling Brisk walking Moderate-intensity physical activity Vigorous-intensity physical activity	79.0	86.2	80.0
	61.3	62.2	61.4
	72.5	75.0	72.9
	33.8	40.9	35.4
Number of people in household (%)			
Living alone Two people Three or more people	4.8	25.5	7.9
	49.4	43.6	48.5
	45.8	30.9	43.5
History of hypertension or metabolic disorders ^a^ (%)	88.9	86.4	88.5
History of cardiovascular disease ^b^ (%)	9.5	6.6	9.1
Depression (CES-D ^c^ ≥ 7 points) (%)	13.6	19.3	14.4
Number of household motor vehicles (%)			
0 1 ≥ 2	2.7	9.9	3.8
	64.4	49.8	34.0
	32.8	40.3	62.2
Body mass index (%)			
< 21 (kg/m^2^) 21– < 25 (kg/m^2^) ≥ 25 (kg/m^2^)	21.1	39.9	24.0
	58.3	46.1	56.4
	20.6	14.0	19.6
Smoking status (%)			
Never Former Current	42.7	91.8	50.2
	40.6	4.9	35.2
	16.7	3.3	14.7
Alcohol drinking habits (%)			
Never Former Current	24.7	67.5	31.2
	5.7	1.2	5.1
	69.5	31.3	63.7
Sleeping hours (%)			
< 7 h 7 to < 8 h ≥ 8 h	39.2	38.3	39.0
	40.0	49.0	41.4
	20.8	12.8	19.6

^a^ History of hypertension, diabetes, hyperlipidemia, or hyperuricemia; ^b^ History of stroke, coronary heart disease, or atrial fibrillation; ^c^ CES-D denotes 11-item Center for Epidemiologic Studies Depression Scale.

**Table 2:** Odds ratios for walking habits ^a^ according to built neighborhood environmental features among retired civil servants, Aichi Worker’s Cohort, 2016.

Leisure-Time Walking More than 3–4 Times/Week						
Environmental Features	No. of Subjects	No. of Cases (%)	Age-Adjusted Model		Multivariable Model ^b^	
			OR (95% CI) ^c^	*p* ^d^	OR (95% CI) ^c^	*p* ^d^
Objective measures						
Walkability						
4–25 26–32 33‒40	526	215 (40.9)	1	***	1	*
	518	214 (41.3)	1.03 (0.81–1.32)		0.96 (0.74–1.24)	
	557	286 (51.4)	1.59 (1.25‒2.03) ^***^		1.37 (1.05‒1.78) ^*^	
Number of parks or green spaces within 500 m radial buffer						
0 1 2–9	658	263 (40.0)	1	***	1	*
	356	165 (46.4)	1.33 (1.02–1.73) ^*^		1.24 (0.94–1.62)	
	587	287 (48.9)	1.46 (1.16–1.82) ^**^		1.33 (1.05–1.68) ^*^	
Number of parks or green spaces within 1000 m radial buffer						
0–1 2–6 7–21	508	204 (40.2)	1	**	1	*^+^*
	573	257 (44.9)	1.24 (0.97–1.58) ^+^		1.12 (0.87–1.44)	
	520	254 (48.9)	1.45 (1.13–1.86) ^**^		1.29 (0.99–1.67) ^+^	
Number of sports facilities within 500 m radial buffer						
0 1 2–10	1112	493 (44.3)	1		1	
	336	151 (44.9)	1.04 (0.81–1.33)		0.96 (0.74–1.23)	
	153	71 (46.4)	1.12 (0.80–1.57)		1.05 (0.74–1.49)	
Number of sports facilities within 1000 m radial buffer						
0 1–2 3–31	563	232 (41.2)	1	**	1	*
	562	240 (42.7)	1.10 (0.87–1.40)		1.03 (0.80–1.32)	
	476	243 (51.1)	1.57 (1.22–2.01) ^***^		1.44 (1.10–1.87) ^**^	
Perceptions						
Good access to public transportation						
Disagree Somewhat agree Strongly agree	218	83 (38.1)	1		1	
	270	109 (40.4)	1.07 (0.74‒1.55)		1.03 (0.71‒1.50)	
	1113	523 (47.0)	1.44 (1.07‒1.94) ^*^		1.31 (0.96‒1.78) ^+^	
Good access to recreation facilities						
Disagree Somewhat agree Strongly agree	422	161 (38.2)	1		1	
	733	324 (44.2)	1.26 (0.98‒1.61) ^+^		1.19 (0.93‒1.53)	
	446	230 (51.6)	1.70 (1.30‒2.23) ^***^		1.60 (1.21‒2.11) ^***^	
Observing others exercising						
Disagree Somewhat agree Strongly agree	287	103 (35.9)	1		1	
	815	349 (42.8)	1.34 (1.02‒1.78) ^*^		1.28 (0.96‒1.70)^+^	
	499	263 (52.7)	1.94 (1.44‒2.62) ^***^		1.83 (1.34‒2.48) ^***^	
Presence of walkable sidewalks						
Disagree Somewhat agree Strongly agree	543	213 (39.2)	1		1	
	581	271 (46.6)	1.35 (1.06‒1.71) ^*^		1.31 (1.03‒1.67)^*^	
	477	231 (48.4)	1.44 (1.13‒1.85) ^**^		1.33 (1.03‒1.72) ^*^	
Poor traffic safety						
Agree Somewhat disagree Strongly disagree	417	179 (42.9)	1		1	
	665	294 (44.2)	1.07 (0.83–1.37)		1.05 (0.81–1.35)	
	519	242 (46.6)	1.16 (0.89–1.50)		1.17 (0.90–1.52)	

*** *p* < 0.001, ** *p* < 0.01, * *p* < 0.05, ^+^ *p* < 0.10; ^a^ Conducting leisure-time strolling or brisk walking more than three‒four times per week; ^b^ Multivariable model adjusted for age, sex, the number of people in household (alone, 2, ≥3 people), body mass index (<21, 21 to <25, or ≥25 kg/m^2^), smoking status (never, former, or current), alcohol drinking habits (never, former, or current), sleeping hours (<7, 7 to <8, or ≥8 h), history of cardiovascular disease (yes, no), presence of hypertension or metabolic disorders (yes, no), depression status (yes, no), and the number of household motor vehicles (0, 1, ≥2); ^c^ OR denotes odds ratio; CI, confidence interval; ^d^ *p* values for trend were calculated using continuous values of objective measures.

**Table 3:** Odds ratios for habitual moderate-intensity physical activity according to built neighborhood environmental features among retired civil servants, Aichi Worker’s Cohort, 2016.

Leisure-Time Moderate-Intensity Physical Activity more than 3–4 Times/Week						
Environmental Features	No. of Subjects	No. of Cases (%)	Age-Adjusted Model		Multivariable Model ^a^	
			OR (95% CI) ^b^	*p* ^c^	OR (95% CI) ^b^	*p* ^c^
Objective measures						
Walkability						
4–25 26–32 33–40	526	128 (24.3)	1	***	1	***
	518	97 (18.7)	0.73 (0.54–0.99) ^*^		0.77 (0.56–1.05) ^+^	
	557	70 (12.6)	0.47 (0.34–0.65) ^***^		0.51 (0.36–0.73) ^***^	
Number of parks or green spaces within 500 m radial buffer						
0 1 2–9	658	145 (22.0)	1	**	1	***
	356	61 (17.1)	0.76 (0.54–1.06)		0.84 (0.59–1.18)	
	587	89 (15.2)	0.64 (0.48–0.86) ^**^		0.68 (0.50–0.93) ^*^	
Number of parks or green spaces within 1000 m radial buffer						
0–1 2–6 7–21	508	116 (22.8)	1	***	1	***
	573	106 (18.5)	0.79 (0.59–1.07)		0.85 (0.63–1.16)	
	520	73 (14.0)	0.57 (0.41–0.78) ^**^		0.62 (0.44–0.88) ^*^	
Number of sports facilities within 500 m radial buffer						
0 1 2–10	1112	217 (19.5)	1	*	1	^+^
	336	60 (17.9)	0.92 (0.67–1.27)		1.00 (0.72–1.39)	
	153	18 (11.8)	0.58 (0.35–0.97) ^*^		0.60 (0.35–1.02) ^+^	
Number of sports facilities within 1000 m radial buffer						
0 1–2 3–31	563	122 (21.7)	1	**	1	*
	562	108 (19.2)	0.92 (0.68–1.23)		0.99 (0.73–1.33)	
	476	65 (13.7)	0.63 (0.45–0.87) ^*^		0.69 (0.48–0.97) ^*^	
Perception						
Good access to public transportation						
Disagree Somewhat agree Strongly agree	218	49 (22.5)	1		1	
	270	52 (19.3)	0.77 (0.49–1.21)		0.81 (0.52–1.27)	
	1113	194 (17.4)	0.72 (0.50–1.02) ^+^		0.78 (0.54–1.12)	
Good access to recreation facilities						
Disagree Somewhat agree Strongly agree	422	79 (18.7)	1		1	
	733	138 (18.8)	0.96 (0.70–1.30)		1.00 (0.73–1.37)	
	446	78 (17.5)	0.88 (0.62–1.25)		0.93 (0.65–1.33)	
Observing others exercising						
Disagree Somewhat agree Strongly agree	287	45 (15.7)	1		1	
	815	150 (18.4)	1.23 (0.85–1.78)		1.29 (0.89–1.88)	
	499	100 (20.0)	1.27 (0.86–1.87)		1.36 (0.91–2.04)	
Presence of walkable sidewalks						
Disagree Somewhat agree Strongly agree	543	112 (20.6)	1		1	
	581	104 (17.9)	0.82 (0.61–1.11)		0.85 (0.62–1.15)	
	477	79 (16.6)	0.75 (0.54–1.03) ^+^		0.80 (0.57–1.11)	
Poor traffic safety						
Agree Somewhat disagree Strongly disagree	417	66 (15.8)	1		1	
	665	131 (19.7)	1.35 (0.97–1.88) ^+^		1.32 (0.95–1.85)	
	519	98 (18.9)	1.23 (0.87–1.75)		1.21 (0.85–1.73)	

*** *p* < 0.001, ** *p* < 0.01, * *p* < 0.05, ^+^ *p* < 0.10; ^a^ Multivariable model adjusted for age, sex, the number of people in household (alone, 2, ≥3 people), body mass index (<21, 21 to <25, or ≥25 kg/m^2^), smoking status (never, former, or current), alcohol drinking habits (never, former, or current), sleeping hours (<7, 7 to <8, or ≥8 h), history of cardiovascular disease (yes, no), presence of hypertension or metabolic disorders (yes, no), depression status (yes, no), and the number of household motor vehicles (0, 1, ≥2); ^b^ OR denotes odds ratio; CI, confidence interval; ^c^ *p* values for trend were calculated using continuous values of objective measures.

**Table 4:** Odds ratios for sports habits according to built neighborhood environmental features among retired civil servants, Aichi Worker’s Cohort, 2016.

Leisure-Time Vigorous-Intensity Physical Activity (Sports) more than 3–4 Times/Week						
Environmental Features	No. of Subjects	No. of Cases (%)	Age-Adjusted Model		Multivariable Model ^a^	
			OR (95% CI) ^b^	*p* ^c^	OR (95% CI) ^b^	*p* ^c^
Objective measures						
Walkability						
4–25 26–32 33–40	526	24 (4.6)	1	*	1	***
	518	36 (7.0)	1.57 (0.92–2.67) ^+^		1.74 (1.00–3.00) ^*^	
	557	39 (7.0)	1.60 (0.95–2.70) ^+^		1.80 (1.03–3.15) ^*^	
Number of parks or green space within 500 m radial buffer						
0 1 2–9	658	29 (4.4)	1	*	1	*
	356	28 (7.9)	1.87 (1.09–3.20) ^*^		2.06 (1.18–3.58) ^*^	
	587	42 (7.2)	1.68 (1.03–2.73) ^*^		1.82 (1.10–3.03) ^*^	
Number of parks or green space within 1000 m radial buffer						
0–1 2–6 7–21	508	22 (4.3)	1	^+^	1	*
	573	36 (6.3)	1.49 (0.87–2.57)		1.53 (0.87–2.67)	
	520	41 (7.9)	1.90 (1.12–3.25) ^*^		2.04 (1.16–3.57) ^*^	
Number of sports facilities within 500 m radial buffer						
0 1 2–10	1112	76 (6.8)	1	^+^	1	
	336	17 (5.1)	0.73 (0.42–1.25)		0.79 (0.45–1.37)	
	153	6 (3.9)	0.56 (0.24–1.31)		0.57 (0.24–1.36)	
Number of sports facilities within 1000 m radial buffer						
0 1–2 3–31	563	31 (5.5)	1		1	
	562	34 (6.1)	1.12 (0.68–1.85)		1.17 (0.70–1.96)	
	476	34 (7.1)	1.34 (0.81–2.23)		1.47 (0.86–2.50)	
Perception						
Good access to public transportation						
Disagree Somewhat agree Strongly agree	218	8 (3.7)	1		1	
	270	16 (5.9)	1.64 (0.69–3.91)		1.66 (0.69–3.99)	
	1113	75 (6.7)	1.89 (0.90–3.99) ^+^		1.84 (0.86–3.91)	
Good access to recreation facilities						
Disagree Somewhat agree Strongly agree	422	15 (3.6)	1		1	
	733	47 (6.4)	1.85 (1.02–3.35) ^*^		1.89 (1.04–3.45) ^*^	
	446	37 (8.3)	2.44 (1.32–4.52) ^**^		2.46 (1.32–4.59) ^**^	
Observing others exercising						
Disagree Somewhat agree Strongly agree	287	10 (3.5)	1		1	
	815	52 (6.4)	1.89 (0.95–3.77) ^+^		1.90 (0.94–3.82) ^+^	
	499	37 (7.4)	2.20 (1.08–4.50) ^*^		2.27 (1.10–4.70) ^*^	
Presence of walkable sidewalks						
Disagree Somewhat agree Strongly agree	543	23 (4.2)	1		1	
	581	41 (7.1)	1.71 (1.01–2.90) ^*^		1.76 (1.03–3.00) ^*^	
	477	35 (7.3)	1.79 (1.04–3.07) ^*^		1.95 (1.12–3.40) ^*^	
Poor traffic safety						
Agree Somewhat disagree Strongly disagree	417	23 (5.5)	1		1	
	665	49 (7.4)	1.37 (0.82–2.28)		1.39 (0.82–2.35)	
	519	27 (5.2)	0.94 (0.53–1.66)		0.94 (0.52–1.68)	

*** *p* < 0.001, ** *p* < 0.01, * *p* < 0.05, + *p* < 0.10; ^a^ Multivariable model adjusted for age, sex, the number of people in household (alone, 2, ≥ 3 people), body mass index (<21, 21 to <25, or ≥25 kg/m^2^), smoking status (never, former, or current), alcohol drinking habits (never, former, or current), sleeping hours (<7, 7 to <8, or ≥8 h), history of cardiovascular disease (yes, no), presence of hypertension or metabolic disorders (yes, no), depression status (yes, no), and the number of household motor vehicles (0, 1, ≥2); ^b^ OR denotes odds ratio; CI, confidence interval; ^c^ *p* values for trend were calculated using continuous values of objective measures.
